# Polycrystal Li_2_ZnTi_3_O_8_/C anode with lotus seedpod structure for high-performance lithium storage

**DOI:** 10.3389/fchem.2023.1135325

**Published:** 2023-05-09

**Authors:** Zhanjun Chen, Tao Wang, Meihuang Liu, Panyu Duan, Feng Xiong, Yang Zhou, Zhenyu Yan, Wei Yang, Han Chen, Zhenyu Yang, Chao Li

**Affiliations:** ^1^ Modern Industry School of Advanced Ceramics, Hunan Provincial Key Laboratory of Fine Ceramics and Powder Materials, School of Materials and Environmental Engineering, Hunan University of Humanities, Science and Technology, Loudi, China; ^2^ School of Materials Science and Engineering, Dongguan University of Technology, Dongguan, Guangdong, China; ^3^ School of Materials and Environmental Engineering, Changsha University, Changsha, China

**Keywords:** lithium-ion battery, anode, lotus seedpod, Li_2_ZnTi_3_O_8_, polycrystalline

## Abstract

Lotus-seedpod structured Li_2_ZnTi_3_O_8_/C (P-LZTO) microspheres obtained by the molten salt method are reported for the first time. The received phase-pure Li_2_ZnTi_3_O_8_ nanoparticles are inserted into the carbon matrix homogeneously to form a Lotus-seedpod structure, as confirmed by the morphological and structural measurements. As the anode for lithium-ion batteries, the P-LZTO material demonstrates excellent electrochemical performance with a high rate capacity of 193.2 mAh g^-1^ at 5 A g^-1^ and long-term cyclic stability up to 300 cycles at 1 A g^-1^. After even 300 cyclings, the P-LZTO particles can maintain their morphological and structural integrity. The superior electrochemical performances have arisen from the unique structure where the polycrystalline structure is beneficial for shorting the lithium-ion diffusion path, while the well-encapsulated carbon matrix can not only enhance the electronic conductivity of the composite but also alleviate the stress anisotropy during lithiation/delithiation process, leading to well-preserved particles.

## 1 Introduction

As an indispensable electrochemical energy storage device, Lithium-ion batteries (LIBs) have been extensively used in various fields, which greatly facilitates our life (Lin et al., 2015). LIBs as power sources for electric vehicles (EVs) and hybrid electric vehicles (HEVs) are further optimized at power density and safety to be highly competitive (Islam et al., 2015; Zhou et al., 2018). Therefore, exploiting electrode materials with high electrochemical performance and security is an urgent goal (Zhang et al., 2018a). However, lithium dendrites are easily produced during charge and discharge for commercial graphite anode, bringing safety risks and not meeting the above requirements (Zhang et al., 2018b).

The spinel Li_2_ZnTi_3_O_8_ (Abbreviated as LZTO) as a promising anode candidate is receiving more and more attention (Hong et al., 2010a), because the LZTO has a higher theoretical capacity than Li_4_Ti_5_O_12_ material and superior safety. Besides, the environmental friendliness and low cost make the LZTO more suitable for large-scale production (Hong et al., 2010b). Unfortunately, the poor electronic conductivity (10^–13^ S m^-1^) and low Li^+^ diffusion coefficient in bulk LZTO are the major obstacles to obtaining unsatisfactory rate performance (Wu et al., 2019). So, researchers have made enormous efforts to improve its electrochemical properties. For example, carbon (Qin et al., 2020; Wang et al., 2021; Bai et al., 2022) or conductive compound (Yang et al., 2017; Yang et al., 2019; Qiu et al., 2021) coating can enhance the surface electronic conductivity and decrease the particle size of LZTO. Metal ion doping (Firdous et al., 2020; Ma et al., 2021; Qi et al., 2021; Tang et al., 2021) can improve the intrinsic electronic conductivity and stabilize the structure of LZTO. Nano-sized LZTO can shorten the diffusion distances of Li^+^ ions (Xu et al., 2013; Wang et al., 2016; Lan et al., 2017).

It is reported that polycrystalline electrode materials, compared with single-crystalline ones, can deliver superior rate performance due to the shorter lithium-ion diffusion pathways (Li et al., 2021; Ryu et al., 2021; Zhang et al., 2021). Nevertheless, the cycling property is unsatisfactory because the stress anisotropy caused by the volume shrinkage and expansion of the primary particles leads to cracking of the material particles, which deteriorates the cycling performance (Singh and Pal, 2020). Moreover, this tendency will be aggravated because there are many defects at grain boundaries in polycrystal particles, such as vacancies, dislocations, and bond deformations, which cause the grains to be in a stress distortion state (Xu et al., 2017; Zhang et al., 2021). Therefore, it is essential to design an ideal structure to alleviate these stress influences. In this paper, we firstly synthesized a polycrystalline Li_2_ZnTi_3_O_8_/C (Abbreviated as P-LZTO) material with a lotus seedpod structure, where the P-LZTO particles are embedded in carbon matrix. This anode material possesses excellent electrochemical properties attributed to the unique design where the polycrystalline structure with few defects at grain boundaries shortens the diffusion distance of lithium-ion in the bulk phase, while the lotus seedpod structure can not only alleviate the negative influences of stress anisotropy but also improve the surface electronic conductivity of these active materials.

## 2 Experimental

### 2.1 Sample synthesis

P-LZTO anode material was synthesized by using molten salt mothed. The typical process as follows: the Zn(CH_3_COO)_2_·H_2_O (commercial, AR), LiOH (commercial, AR), TiO_2_ (commercial, AR), C_6_H_12_O_6_ (commercial, AR), NaCl (commercial, AR), and KCl (commercial, AR) were mixed (molar ratio 1:2:3:1.5:10:10) and grounded for 1 h with planetary ball mill. The mixture was then placed in a corundum crucible and annealed at 600°C for 24 h under N_2_ atmosphere. The product was collected after naturally cooling the furnace to room temperature and then washed and centrifugated several times with distilled water until a silver nitrate solution detected no free chloride ions. The product was finally dried at 80°C for 12 h.

### 2.2 Sample characterization

The compositions were tested by X-ray powder diffraction using Cu-Kα radiation (XRD, *λ* = 1.54056 Å, Bruker D8) from 10° to 90° with a step size of 0.02°s^-1^. And the crystal structure was further analyzed by Raman spectrum (LabRAM HR800) with a helium laser (*λ* = 633 nm) in the wave number range of 100-2000 cm^-1^. The chemical states at the surface of the samples were obtained from *X*-ray photoelectron spectroscopy (XPS, Thermo escalab 250XI) measurement. The morphology was characterized using a scanning electron microscope (SEM, Philip-XL30) and transmission electron microscopy (TEM, Tecnai G2 F30). The thermal behaviors of the P-LZTO were characterized by thermogravimetric (TG) and differential thermal analyses (DTA) in air from room temperature to 800°C using a thermal analyzer (TA Instruments Q500) at a heating rate of 10°C min^-1^.

### 2.3 Electrochemical measurement

The electrochemical properties were characterized in two-electrode half cells (CR 2025) where the Li foil was used as the counter electrode. The working electrode was fabricated by coating and pressing a mixture of P-LZTO power as active material, conductive carbon black as a conductive additive, and polyvinylidene fluoride as the binder (8:1:1 in mass ratio) with N-methylpyrrolidone on Al foil circular flakes. The cells were assembled in a glovebox (O_2_ and H_2_O levels <1 ppm) using Metallic lithium foil and a micro-porous polypropylene membrane (Celgard 2400) as the negative electrode and the separator, respectively. The electrolyte was 1 mol L^-1^ LiPF_6_ in a 7:3 (v/v) mixture of dimethyl carbonate (DMC) and ethylene carbonate (EC). The charge-discharge curves were recorded at designed current densities in the voltage range of 0.02–3.0V (vs. Li^+^/Li) on a CT 2001A cell test instrument (Land Electronic Co.). The electrochemical impedance spectroscopy (EIS, PGSTAT302N) was conducted to check impedance with an AC voltage of 0.02V amplitude in the frequency range of 50 mHz–10^5^ Hz. Cyclic voltammetry (CV) measurements were tested on the CHI660E electrochemical workstation at a potential range of 0.02–3 V. Before the SEM and TEM analyses of the electrodes after cycle testing, the electrodes were manipulated as follows: the test cells were disassembled carefully and washed with 1-methyl-2-pyrrolidone ultrasonically several times to remove PVDF binder, then, the collected solid particles were dried at 100°C in an oven.

## 3 Results and discussion

**FIGURE 1:**
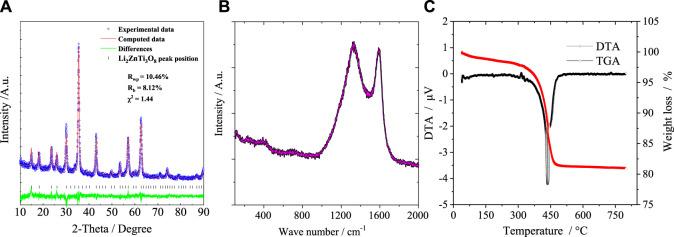
XRD pattern and Rietveld refinement **(A)**, Raman spectrum **(B)** and TG-DTA curves **(C)** of the P-LZTO sample.

The XRD pattern of the as-synthesized P-LZTO is shown in Figure 1A. All the diffraction peaks can be indexed to a cubic structure with space group P4_3_32 (JCPDS card No.86-1512) with no impurities detected. To understand the detailed crystal structure of P-LZTO, Rietveld refinement based on the recorded XRD data was performed. As listed in Table 1, the profile R-value (R_p_) and weighted-profile R-value (R_wp_) indicate that the refinement results are acceptable. And the cell parameters *a* and *V* are 8.3809 Å and 536.38 Å^3^, respectively, which agrees with the standard parameter (*a* = 8.371 Å) derived from the JCPDS card No.86-1512. The mean grain diameters calculated by the Scherrer equation *D* = *Kλ*/(*β*cos*θ*) based on the (311) plane is 15.4 nm. Raman spectrum was acquired for the P-LZTO sample to identify glucose-derived carbon’s existence and graphitization state. In the Raman spectrum, besides the vibrations associated to LZTO (below 800 cm^-1^), the peaks at ca. 1325 cm^-1^ and at ca.1586 cm^-1^ could be assigned to D bond and G bond in carbon, associating with amorphous and graphitized carbon, respectively. The intensity ratio of the D band and G band (*I*_*D*_/*I*_*G*_) could be calculated as 1.05, indicative of the high electronic conductivity of residual carbon (Ren et al., 2016a; Ren et al., 2016b) which means a relatively high graphitization degree of the carbon derived from glucose. The content of carbon was also carried out by TG-DTA in air from 25°C to 800°C as shown in Figure 1C. Clearly, a weight loss of 2% below 300°C can be attributed to the vaporization of the absorbed water. There is a large weight loss about 16.5% accompanying a strong exothermic peak between 300°C and 500°C due to the vigorous combustion reactions of the residual carbon. No weight loss and thermal peaks were observed above 500°C. Therefore, the content of carbon for P-LZTO sample is about 16.5%.

**TABLE 1:** Structural parameters of the P-LZTO sample by the XRD Rietveld Refinements (Space group *P4*_*3*_ *32*, *a* = *b* = *c* = 8.371 Å, *α* = *β* = *γ* = 90°).

*a* (Å)	Volume (Å^3^)	Atom	Wyckoff position	*x*	*y*	*z*	Occupancy	R_p_ (%)	R_wp_ (%)
8.3809	536.38	Li1	8c	0.9984	0.9984	0.9984	0.5	8.12	10.46
		Zn	8c	0.9984	0.9984	0.9984	0.5		
		Li2	4b	0.6250	0.6250	0.6250	1		
		Ti	12d	0.3677	0.8823	0.1250	1		
		O1	24e	0.1050	0.1280	0.3920	1		
		O2	8c	0.3920	0.3920	0.3920	1		

**FIGURE 2:**
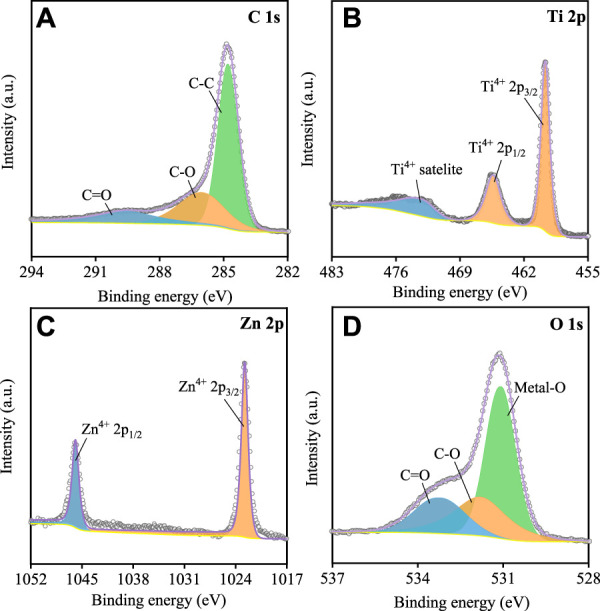
XPS spectra of the P-LZTO sample: **(A)** C1s, **(B)** Ti2p, **(C)** Zn2p, **(D)** O1s.

XPS measurement further determined the chemical states at the surface of the sample. It can be seen that the high-resolution spectrum of C1s (Figure 2A) was deconvoluted into three peaks around 284.8, 286.1, and 289.7 eV corresponding to C-C, C-O, and C=O bonds, respectively, which is consistent with other studies (Qin et al., 2020). Usually, there are many defects at grain boundaries in polycrystal particles, such as vacancies, dislocations, and bond deformations, which might cause the transformation for the valance states of Ti, Zn, and O elements (Fan et al., 2020; Ryu et al., 2021). Here, the high-resolution spectrum of Ti2p (Figure 2B), Zn2p (Figure 2C), O1s (Figure 2D) were displayed. As can be seen that no spectra were observed except the peaks around 465.3 eV and 459.6 eV, which belong to the Ti 2p_1/2_ and Ti 2p_3/2_ of Ti^4+^ in P-LZTO (Figure 2B), respectively. Similarly, only the peaks of Zn^2+^ (1022.7 eV and 1045.8 eV belong to Zn 2p_3/2_ and Zn 2p_1/2_, respectively) can be observed in the spectrum of Zn2p (Figure 2C). Besides, in the high-resolution spectrum of O1s (Figure 2D), the peaks around 533.27 eV, 531.81 eV, and 531.12 eV can be ascribed to C=O, C-O, and Metal-O, respectively. Therefore, from the analysis results of XPS spectra and XRD pattern, it can be concluded that P-LZTO samples show a perfect crystal structure, and almost no defects like vacancies can be observed in its crystal structure.

**FIGURE 3:**
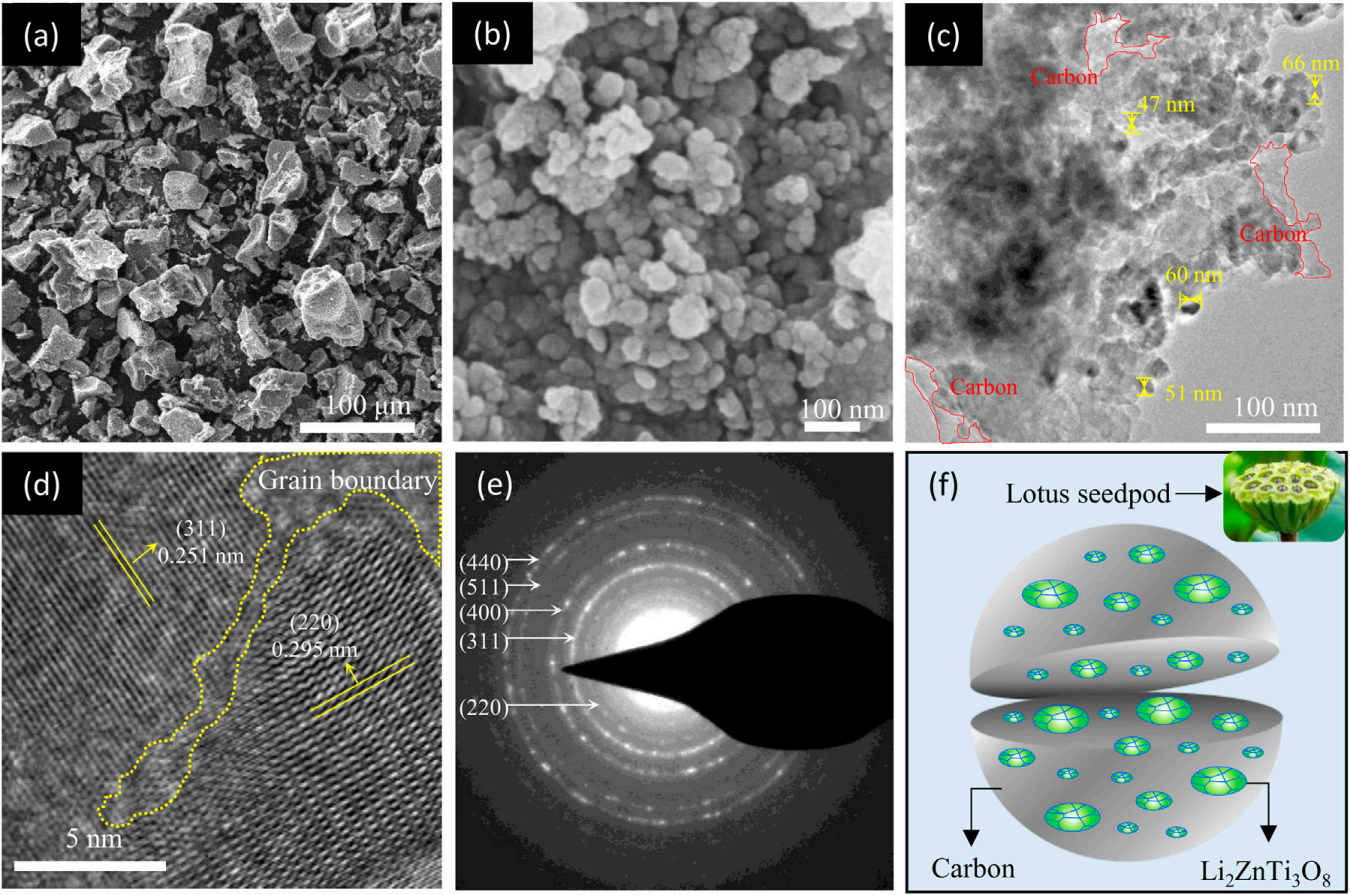
**(A)** Low- and **(B)** high-magnification SEM images of the P-LZTO sample; **(C)** TEM, **(D)** HRTEM images and **(E)** their corresponding SAED pattern of the P-LZTO sample; **(F)** is the schematic drawing about the structure of the P-LZTO sample according to the results of SEM and TEM tests.

The as-synthesized P-LZTO sample has excellent powder fluidity, which is often related to the material’s microstructure. Therefore, the morphology of the P-LZTO sample was analyzed by SEM and TEM measurements. From the SEM images in Figures 3A,B, the secondary particles composed of many primary particles with a diameter of tens of nanometers are uniformly dispersed. TEM images (Figure 3C) further showed that a large number of nanoparticles were embedded in carbon materials derived from the cleavage of glucose under high temperatures. In other words, many nanoparticles were wrapped by continuous carbon materials to form secondary particles. Moreover, it can also be observed from the HRTEM image (Figure 3D) that there are two different lattice fringes in a nanoparticle: the lattice fringes with a distance of 0.251 nm along the (311) crystal plane while the lattice fringes with a distance of 0.295 nm corresponding to the (220) crystal face, and a grain boundary is formed between the two grains, reflecting typical polycrystalline structure. This conclusion is further confirmed in the SAED pattern in Figure 3E, where a lot of diffraction rings can be observed, demonstrating its good crystalline feature, which is a typical characteristic of polycrystalline structure (Xu et al., 2020; Ye et al., 2020). Based on the results of SEM and TEM analysis, a schematic diagram of the structure of P-LZTO sample was illustrated in Figure 3F. The structure of the synthesized P-LZTO sample is similar to the lotus seed: the polycrystalline P-LZTO nano-particles are embedded in carbon material, which is beneficial to enhance the electronic conductivity for the active material, and alleviate the negative influences of stress anisotropy in the process of charging and discharging.

**FIGURE 4:**
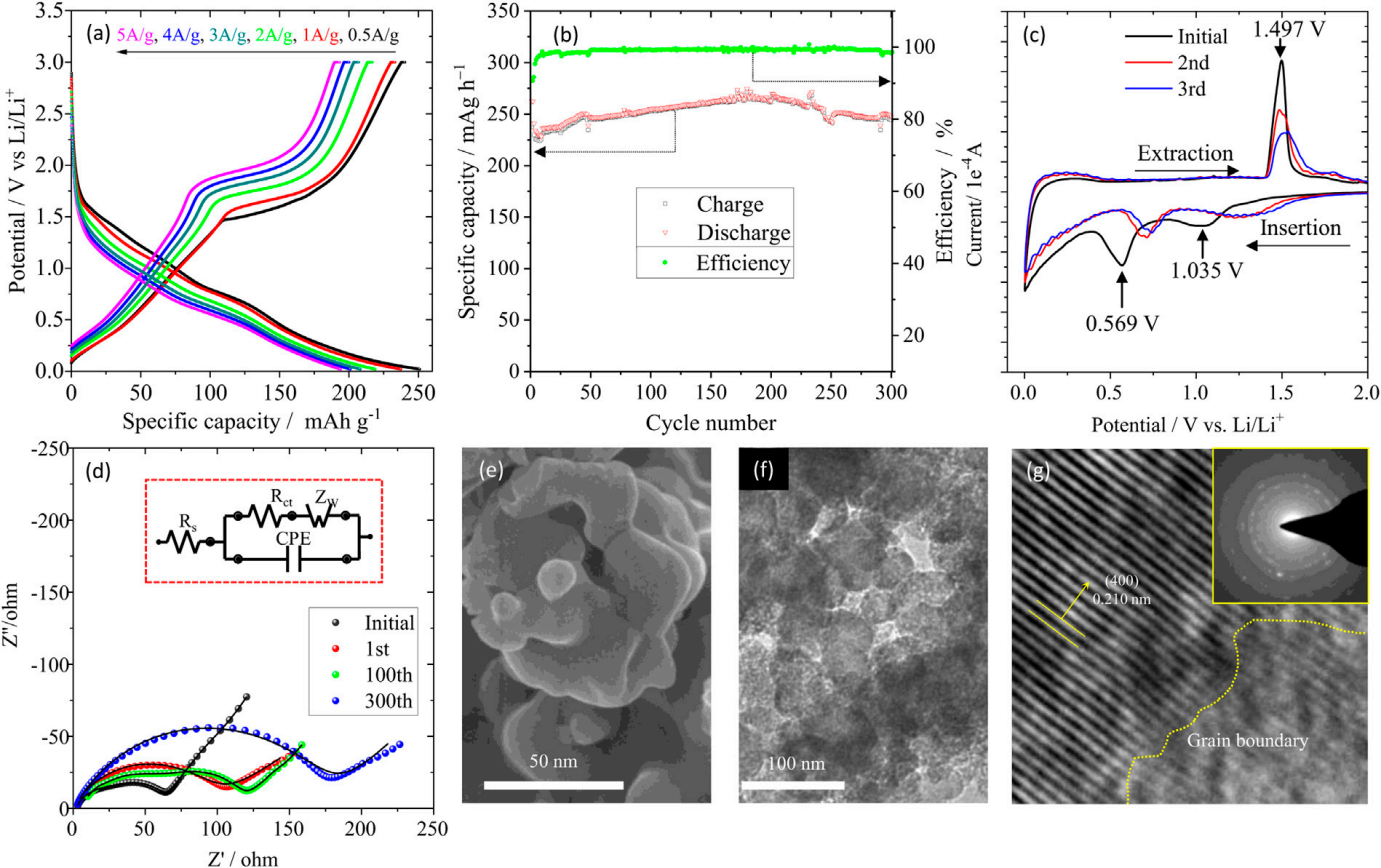
Rate capability **(A)**, cycle performance **(B)** and CV curves **(C)** of the P-LZTO sample; EIS results **(D)** for P-LZTO after designed cycling test; SEM **(E)**, TEM **(F)** and HRTEM **(G)** images of the electrode after 300th cycle; the inset part in **(D)** and in **(G)** are the equivalent circuit and the corresponding SAED pattern, respectively.

Rate performance was tested at 0.5, 1.0, 2.0, 3.0, 4.0 and 5.0 A/g each for 5 cycles and the representative charge/discharge curves were depicted in Figure 4A. The sample delivered a specific charge capacity of 240.3, 233.2, 216.8, 207, 200.1 and 193.2 mAh g^-1^ at 0.5, 1.0, 2.0, 3.0, 4.0 and 5.0 A/g, respectively. These results are superior to the previously reported (Wu et al., 2019; Qin et al., 2020) values where the specific capacities were ∼140 mAh g^-1^ when the current density of charging was 1.6 A/g. Galvanostatic charge-discharge for P-LZTO was conducted at 1.0 A/g between 0.02 and 3.0 V (vs Li/Li^+^) (Figure 4B). It can be observed that the initial coulomb efficiency is as high as 90.7%, which means that only a thin solid electrolyte interphase (SEI) film is formed on the surface of P-LZTO particles, benefiting to obtaining excellent rate performance (in Figure 4A). The charge/discharge capacities are 241.4/245.3 mAh g^-1^ after 300 cycles for P-LZTO electrode, revealing that this anode material also exhibits excellent cycling performance. The reason of a sluggish increase for charge/discharge capacities in the first 200 cycles may be mainly attributed to some side reactions for solid electrolyte interface formation composed of organic lithium alkylcarbonates, lithium adsorption in the conductive additive carbon black, and irreversible electrochemical decomposition of the electrolyte, and so on, which is also observed in previous studies (Wu et al., 2019). For better understanding the electrochemical reactions involved, CV profiles were measured for the fresh cell (Figure 4C). In the first cycle, the cathodic/anodic peaks at ca.1.035/1.497 V are attributed to the Ti^4+^/Ti^3+^ redox couple, while the cathodic peak around 0.569 V to the generation of SEI films and those below 0.5 V to the transition from ordered rock-salt structure to disordered quasi-rock-salt structure (Yang et al., 2019). From the second cycle, the CV plots superpose very well, indicating good reversibility of Li^+^ intercalation and deintercalation in the P-LZTO anode. Meanwhile, the potential variance of the cathodic and anodic peak decreases from 0.457 V for the 1^st^ cycle to 0.21 V for the latter cycle, demonstrating the weakened polarization for the P-LZTO anode. To understand the electrochemical kinetics in the P-LZTO electrode, EIS was employed to test the interface reaction behaviors between electrolyte and electrode before and after designed cycling. As depicted in Figure 4D, the Nyquist plots are comprised of one semi-circle at high-frequency ranges and a small tail at low frequency, which is attributed to charge transfer resistance and lithium-ion diffusion, respectively. It can be seen that the charge transfer resistance increases slightly during the cycling process, indicating that the interface of the electrode-electrolyte can maintain stability. Moreover, the Li-ion diffusion coefficient ( $D_{Li^{+}}$ ) in the P-LZTO electrode was calculated by Eq. 1 where *T* (298K) is the absolute temperature in the experiment, *F* (96500 C/mol) is the Faraday constant, *R* (8.314 J/(molK)) is the gas constant, *n* (1 mol) is the number of electrons per molecule that participates in the electron transfer reaction, *A* (0.785 cm^-2^) is the surface area of the electrode, and *C* (0.001 mol/cm^-3^) is the concentration of Li ions in the P-LZTO electrode. The *σ* is obtained from the slope of a plot of Z′ against ω^-0.5^ according with Eq. 2. As shown in Table 2, it can be found that the difference between the value of $D_{Li^{+}}$ after 1^st^ charge and 300^th^ charge is quite small, indicating that the crystal structure is stable. To further verify the structural stability of P-LZTO during the cycling process, the cell was disassembled after 300^th^ cycle, and the active material was analyzed by SEM and TEM measurements. As shown in Figure 4E, the original morphology of the sample can still be maintained after 300^th^ cycling, and no cracks caused by stress anisotropy of polycrystalline structure were found, which is common in other polycrystalline materials such as LiNi_x_Co_y_Mn_1-x-y_O_2_ (Liu et al., 2022). TEM image (Figure 4F) also shows that the P-LZTO particles with intact morphology are embedded in the carbon matrix. They maintain good interface compatibility, just like the morphology before cycling observed in Figure 3C. Moreover, from the HRTEM image (Figure 4G) and the corresponding SAED pattern, it can be seen that the lattice fringes are clearly visible. The grain boundary is continuous and intact for the polycrystalline structure. In conclusion, the P-LZTO anode shows excellent rate and cycling performance, because the polycrystalline structure could shorten the diffusion distance of lithium ion in bulk phase, and its perfect crystal structure and Lotus seedpod structure can alleviate the negative influences of stress anisotropy which can cause cracking and powderization of active material particles during charge/discharge cycles.

$$D_{Li}=\frac{R^{2}T^{2}}{2A^{2}n^{4}F^{4}C^{2}\sigma^{2}}$$ (1)

$$Z^{\prime }=R_{S}+R_{ct}+\sigma \omega^{-1/2}$$ (2)

**TABLE 2:** Li^+^ diffusion coefficients after cycling based on EIS tests.

Samples	1^st^ charge	100^th^ charge	300^th^ charge
$D_{Li^{+}}\left[cm^{2}\cdot s^{-1}\right]$	2.33 × 10^−11^	1.55 × 10^−11^	1.25 × 10^−11^

Figure 4 Rate capability a), cycle performance b) and CV curves c) of the P-LZTO sample; EIS results d) for P-LZTO after designed cycling test; SEM e), TEM f) and HRTEM g) images of the electrode after 300th cycle; the inset part in d) and in g) are the equivalent circuit and the corresponding SAED pattern, respectively.

## 4 Conclusion

The P-LZTO anode materials with the polycrystalline structure were first prepared using the molten salt method. The P-LZTO sample is shaped like a lotus seed, with many nanoparticles with polycrystalline structure, no defects and pure phase are uniformly embedded in the carbon materials to form secondary particles. The electrochemical performance test results show that the P-LZTO anode materials, not like other polycrystalline ones, exhibit excellent rate and cycle performances attributed to the unique lotus seed structure where the carbon materials are beneficial to enhance the electronic conductivity and alleviate the negative influences of stress anisotropy during the process of charging and discharging. Meanwhile, the polycrystalline structure can shorten the diffusion distance of lithium ion in bulk phase. The SEM and TEM analysis results for the electrode material after 300 cycling proved that the P-LZTO material can maintain the morphology and structure stable before and after cycling, indicating that the lotus seed structure can inhibit cracking and powderization of active material particles during charge/discharge cycles. Therefore, the method for designing a lotus seed structure for polycrystalline material might provide a new idea for improving the electrochemical properties of other electrode materials.

## Data Availability

The raw data supporting the conclusion of this article will be made available by the authors, without undue reservation.
